# Product diffusion through on-demand information-seeking behaviour

**DOI:** 10.1098/rsif.2017.0751

**Published:** 2018-02-21

**Authors:** Christoph Riedl, Johannes Bjelland, Geoffrey Canright, Asif Iqbal, Kenth Engø-Monsen, Taimur Qureshi, Pål Roe Sundsøy, David Lazer

**Affiliations:** 1 Northeastern University, Boston, MA, USA; 2 Harvard University, Cambridge, MA, USA; 3 Telenor Research, Oslo, Norway

**Keywords:** diffusion, computational social science, social networks, complex systems

## Abstract

Most models of product adoption predict S-shaped adoption curves. Here we report results from two country-scale experiments in which we find *linear* adoption curves. We show evidence that the observed linear pattern is the result of active information-seeking behaviour: individuals actively pulling information from several central sources facilitated by modern Internet searches. Thus, a constant baseline rate of interest sustains product diffusion, resulting in a linear diffusion process instead of the S-shaped curve of adoption predicted by many diffusion models. The main experiment seeded 70 000 (48 000 in Experiment 2) unique voucher codes for the same product with randomly sampled nodes in a social network of approximately 43 million individuals with about 567 million ties. We find that the experiment reached over 800 000 individuals with 80% of adopters adopting the same product—a winner-take-all dynamic consistent with search engine driven rankings that would not have emerged had the products spread only through a network of social contacts. We provide evidence for (and characterization of) this diffusion process driven by active information-seeking behaviour through analyses investigating (a) patterns of geographical spreading; (b) the branching process; and (c) diffusion heterogeneity. Using data on adopters' geolocation we show that social spreading is highly localized, while on-demand diffusion is geographically independent. We also show that cascades started by individuals who actively pull information from central sources are more effective at spreading the product among their peers.

## 1. Introduction

Social influence plays a prominent role across many social sciences, for example in the study of contagion in sociology [1], social learning in problem-solving [2], herding behaviour in economics [3], price bubbles in financial markets [4] and well-being in public health [5–7]. Regarding economic outcomes such as product adoption [8,9], social influence plays an especially important role in markets where attitudes and tastes are influenced by other individuals. This can lead to widespread diffusion and popularity of some products, but not others [10]. Economic outcomes are also affected when people passively receive information about products from a central source, such as television or radio advertising.

The rise of the Internet has changed the way information diffuses. Previously, only a few central broadcast sources (such as radio or TV stations) could broadcast information about products, but now ordinary individuals can post information online to be discovered by prospective adopters proactively searching and discovering information. This is social influence—but influence among strangers, mediated by two steps: (i) posting by the source, and (ii) searching by the seeker. More generally, in recent history we have witnessed the emergence of a novel diffusion mechanism, where people actively seek information that is facilitated by online search tools (such as Google) and search functionality within social media sites (such as Twitter or Facebook) [11,12]. However, behaviour change through active information-seeking behaviour has not been systematically investigated and is not well understood. Moreover, diffusion processes often mix and interact [11,13,14] in unknown ways, leading to complex spreading patterns [15].

Better understanding of on-demand information-seeking behaviour and mixing with social diffusion can inform our understanding of important aspects of diffusion processes on networks that are not well understood [9,15]. Recent research suggests that central sources or opinion leaders have limited effect on diffusion processes [16,17]. However, if a diffusion process is governed by active searching behaviour, central sources may play an important role: Even though central sources may have limited influence to *push* information to passive recipients, they can play influential roles when individuals actively *pull* information. Combined with popularity and ranking effects produced by search engines (which have been investigated separately; see [18]), central sources can thus dramatically shape diffusion processes. As a result, if the prevalent diffusion is on-demand, this may result in near-linear product adoption rates, driven by an underlying constant rate of interest in the product rather than the typical S-shaped curves predicted by social diffusion [8]. However, depending on the presence and relative proportion of peer-to-peer sharing and the strength of on-demand diffusion (ODD) signified by the underlying rate of search for the product, we may expect a concave diffusion curve. The exact shape of the diffusion curve would depend on the mixing proportion of the two regimes and the fitness with which the product spreads overall.^1^

Several other aspects become apparent when considering a diffusion process that is strongly driven by ODD. First, we would expect the absence of geographical clustering of product adopters. Second, we may see winner-take-all dynamics among equivalent products that are shaped by search engine rankings as interested individuals all discover and adopt the same product, compared with a more equal distribution across products when product adoption spreads exclusively along social ties. That is, once product adoption is driven by on-demand information seeking rather than peer-to-peer, individuals are presented with a similarly ranked list through search engines and Top 10 pages, that disproportionately rewards products at the top of the list [19,21]. Finally, better understanding of product diffusion driven by active information-seeking behaviour may lead to important insights into homophilous network connections. These insights may provide a basis to better predict cascade sizes and improve targeting [22].

In this paper, we report the results from two country-level experiments in which we quantify and characterize the adoption of a new product. We track the adoption through 70 000 unique voucher codes (48 000 codes in Experiment 2) resulting from active information-seeking behaviour and quantify the mixing between this ODD process and peer-to-peer diffusion as they occur simultaneously. Our experiments are among the largest social science experiments ever conducted, resulting in product adoption by over 1 million customers across both experiments. We provide evidence for (and a direct comparison of) product adoption processes driven by posting/searching behaviour and peer-to-peer diffusion. We show how active information-seeking behaviour shapes product adoption curves leading to dynamics that are consistent with those produced by search engines rankings and can lead to winner-take-all dynamics that are distinctly different from those expected if products would diffuse purely from peer-to-peer.

There is an extensive body of literature on information diffusion [3,23–26], cascade sizes [22], two-step diffusion [11,13,14,27,28], product adoption [8,20,29–32] and diffusion under external influences [12]. With some exceptions [5,7,10,33–35], most studies of diffusion processes rely on observational data—which generally confound homophily and contagion [36]. Our paper differs fundamentally from the existing empirical literature studying diffusion processes. The existing research uses observational data in which confounding effects can always be present [36]. Instead, we seed information about a new product with randomly selected individuals, thus avoiding confounds. In contrast with studies driven by observational data, all voucher codes refer to the same product, avoiding confounds with product differences. Much research on diffusion focuses on the information flow on social media sites such as Twitter or Facebook [22,27,37]. Here, we study behaviour change: the adoption of a product. Findings that hold in the case of information diffusion may be different in the case of behaviour change [38].

Onnela and Reed-Tsochas' work on the adoption of Facebook applications (‘apps’) [20] is most similar to our research, but it cannot cleanly distinguish product attributes and diffusion processes—here, we study adoption of one product tracked using many unique codes. The study by Goel *et al*. [28] is also closely related to ours in its focus on small and big online diffusion events using a comprehensive dataset from Twitter, but it also studies the diffusion of heterogeneous items. Further, it does not touch on the role of on-demand information-seeking behaviour in shaping diffusion events. Instead, it examines popularity gained through a single, large broadcast. It analyses a weaker version of social influence (information sharing) rather than stronger behaviour change such as the adoption of a new product. Work by Karsai *et al*. [9,39] investigates how spontaneous adopters arriving at a constant rate affect the social spreading of a single product. Our analysis builds on and extends this research by quantifying and characterizing patterns in product adoption that are driven by on-demand information-seeking behaviour, the geographical distribution of adopters, cascade sizes, and the competition between products that spread simultaneously.

## 2. Research design

To study the interplay of social and non-social diffusion processes in product adoption, we designed two country-scale network experiments around a marketing campaign. In our marketing experiments we partnered with a local mobile phone operator to target 70 000 (48 000 in Experiment 2) randomly sampled individuals through mobile phone text messages, with 70 000 (48 000) unique voucher codes offering free data traffic for their cellphone plans (see electronic supplementary material for details of the experimental design and how seed customers were selected). Each voucher code referred to the same product—a one-time allowance of 15 MB (60 MB in Experiment 2) of data traffic—yet allowed us to trace many diffusion cascades that were unfolding simultaneously. Individuals exposed to a voucher code could (a) redeem the voucher code themselves (i.e. adopt the product) and (b) *independently of redeeming the voucher themselves,* pass the code on to others. That is, each voucher code uniquely identifies a diffusion process from a randomly targeted individual and how it spreads to a potentially large number of other individuals. While each code could be adopted an unlimited number of times, every individual was only allowed to adopt one code (i.e. redeem only one voucher code from the campaign). Individuals who redeemed a voucher code were black-listed and not eligible to redeem another from the same campaign. Redeeming a voucher code effectively conveyed full ‘immunity’ to any other code. Thus, the experiment allowed us to study the simultaneous diffusion of 70 000 (48 000) competing codes referring to the same underlying new-to-the-world product.

After seeding all voucher codes simultaneously in the network, we observed the adoption of each code over a period of two weeks. Using call detail records from the network of the operator we partnered with, we constructed the complete social graph of 43 million individuals (nodes) and 567 million connections between individuals (edges) to study how voucher codes spread through the network of existing social ties (see electronic supplementary material). For each voucher code, we constructed an invasion tree in which every adopter of the code is linked with prior adopters in the individual's own social network. The social network constructed from call detail records is appropriate to study diffusion for two reasons. First, prior research has demonstrated that phone records can be used to construct valid and high-quality social networks [40]. Second, voucher codes were received via SMS text messages and could be forwarded via SMS text messages. Since voucher codes consisted of random alphanumeric sequences, error-free verbal transmission was cumbersome. Transmission via SMS was the predominant pathway through which voucher codes were passed on.

The nature of our research design (randomly seeded unique codes, referencing the same novel product) offers several advantages that would make the interpretation of causal processes challenging when relying only on observational data. First, voucher codes were seeded with randomly sampled nodes in a network of existing social ties (see electronic supplementary material for details on the experimental protocol). Thus, the starting position for any one code was uncorrelated with endogenous aspects of the seed's position in the social network or the characteristics of the individual receiving the seed product code. Each code should have the same chances of adoption and diffusion. Second, studies on new product diffusion have typically used only aggregated data on (overall) product sales, rather than the individual-level observations that our experimental design makes possible [30]. Third, our research design employed functionally identical products, each represented by different voucher codes. In related work studying the spreading of memes on Twitter [26] or apps on Facebook [20], all products were different and product selection effects were known to exist. In particular, individuals are more likely to share better products [31]. Our research design allows us to disentangle the spreading process from the nature of the product. Thus, we do not simply observe the product diffusion and adoption process that happens. Instead, we deliberately intervene in the data generating process to collect data on independent spreading processes that are independent of node locations in the network (because of the random assignment), are not conditional on spreading, and are not confounded with product differences. This allows us to gain better understanding of human behaviour related to diffusion and contrasts with studies driven by observational data, in which confounding effects can always be present [36].

## 3. Results

**Figure 1.**
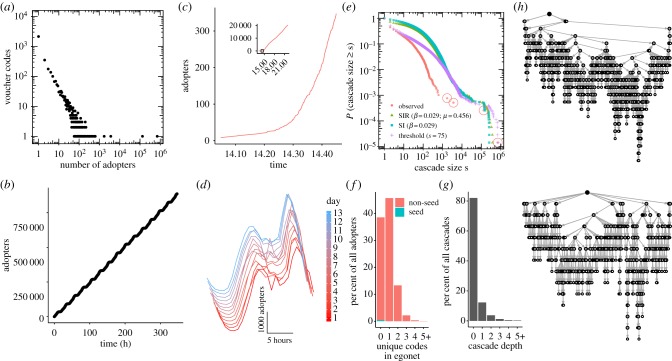
Adoption and diffusion of codes in a social network. (*a*) Number of adopters for each unique code on log-log scale. (*b*) Cumulative number of adopters across all codes aggregated at the full hour. (*c*) Adoption of the most popular code. Inset: After 10 h, the code had been adopted by more than 20 000 adopters, then adoption continued in a linear fashion to more than 700 000 adopters over the next 13 days (not shown). Main: Zooming into the beginning of the linear adoption period (45 min). (*d*) Repeating daily patterns of similar shape (all codes). The minimum number of daily new adopters was around 04.00 with an average of 46 hourly adopters compared with a peak at 19.00 with an average of 5479 adopters (RMSE across all possible day-wise comparisons: 1619.89). (*e*) Complementary cumulative distribution of code popularity for simulation of SIR, SI, and threshold models and observed spreading for cascades with at least one adopter. Neither of the canonical diffusion models provides a good explanation of the skewed popularity of the most popular code. The models instead predict more codes would be adopted by at least 1000 adopters than observed. The SIR model predicts that, on average, 54 codes should accumulate more than 1000 adopters, compared with just four (highlighted) that were observed in the experiment. (*f*) Distribution of distinct voucher codes in adopters' ego networks at the point of adoption. Those with no prior adopters likely adopted via on-demand diffusion from a central source (except the small proportion of adopters who were original seeds). The majority of adopters had exactly one prior adopter in their ego network, which indicates the direct peer-to-peer spreading process. Sixteen per cent of adopters had two or more unique codes in their social network, which indicates competition among codes. (*g*) Distribution of cascade depths. (*h*) Invasion trees of the two largest cascades. Seed node shown in black (top), leaves in white, other nodes in grey.

The overall adoption activity reveals several notable features (figure 1*a*; see electronic supplementary material for detailed analyses of Experiment 2). First, we find large heterogeneity of outcomes. Most codes do not spread at all, while some spread to a substantial number of individuals, starting from a single seed. Of the initially seeded 70 000 codes, only 3886 were adopted by at least one individual (6% of codes). Second, codes were ultimately adopted by a total of 886 025 individuals—which corresponds to an average of 12.66 adopters per the total number of codes in the study, and an average of 228.01 adopters per adopted code. Third, the distribution of the number of adopters per code is heavy-tailed. Third, the distribution of the number of adopters per code is heavy-tailed. Remarkably, the most popular code accounts for almost 80% (704 466) of all adopters. Only three other codes were adopted by more than 1000 individuals, the rest acquired significantly fewer adopters. While we observe a lot of overall diffusion in terms of the final number of individuals reached—relative to the total number of seeds—most codes did not spread at all. Fourth, the overall spreading follows a linear pattern over time—not the typical S-curve one would expect if diffusion was driven by a social spreading process (figure 1*b*).

However, there is substantial variation among the shapes of the diffusion curves of individual codes (see electronic supplementary material). The most popular code spread in a linear fashion, adding adopters at an almost constant rate of 48 686 per day on average (excluding the incomplete first and last days; s.d.: 2903; figure 1*c*, inset). In fact, 4749 individuals adopted the most popular code instead of the code they were seeded with. Assuming a social spreading process, this linear spreading is unexpected. The transition from exponential growth to the linear pattern happened within a short time window after the launch of the experiment. Within 96 min, the code moved from under 10 adopters per 6-min time window to over 200 (we confirm a structural change in the time series at *p* < 0.001; see electronic supplementary material). Fifth, the spreading process followed distinct diurnal cycles (following an oscillating 24 h curve). The patterns were extremely similar across the days of the experiment (figure 1*d*). The minimum number of daily new adopters occurred at around 04.00 with only 46 hourly adopters, compared with a peak at 19.00 with an average of 5479 adopters.

To compare the observed diffusion process to the pattern expected in the case of pure social diffusion, we simulate different stochastic individual contact models: susceptible–infected (SI), susceptible–infected–recovered (SIR), and a threshold model [41,42]. We simulate the diffusion processes on the full observed social network (34M nodes, 567M edges), seeding the same number of codes with the same nodes in the network (figure 1*e*; the electronic supplementary material provides additional robustness tests using different model parameters, seeding procedures and networks that include additional random ties). The simulation shows that (a) the observed spreading pattern cannot be explained by a pure social diffusion process and (b) if the diffusion was only driven by social spreading, the diffusion process would have resulted in codes being adopted more equally. This points to some additional, non-social spreading process that shaped the observed diffusion outcomes, leading to a skewed distribution of code adoptions and creating an unequal distribution in which one code got adopted significantly more often than any other.

To examine a possible transition from social spreading to other diffusion modalities, we first examined the number of prior adopters in an adopters' social network. When a voucher code appeared at a node in the network that has no connections to other nodes that have previously adopted the code, that appearance can only be explained by the influence of some unobserved exogenous source (or via social ties that are not part of our observed social network constructed from phone records, a possibility we investigate in the next paragraph). We indeed do observe that a large portion of codes spread via non-social diffusion to individuals who had no connection to prior adopters. About 37% of all adopters had no prior adopters in their social network, but only a tiny fraction of them (1%) received a seed code (figure 1*f*). Where did these individuals get the voucher codes from? As outlined in the introduction, one possibility is that they actively searched for a ‘free data’ voucher on the Internet. Indeed, we confirm that several voucher codes have been posted online on message forums (see electronic supplementary material). A systematic online search for all voucher codes in Experiment 2 revealed that every code with more than 1000 adopters was posted online and discoverable via Google. Therefore, we have evidence that despite the peer-to-peer nature of the network in which the codes were seeded, codes also spread outside social networks via alternative pathways. Furthermore, we find significant competition among codes, with 16% of individuals having two or more unique codes in their social network (figure 1*f*). Most invasion trees are shallow, spanning only a few generations from the source of the cascade (figure 1*g*). We show example invasion trees of the two largest cascades in figure 1*h*.

**Figure 2.**
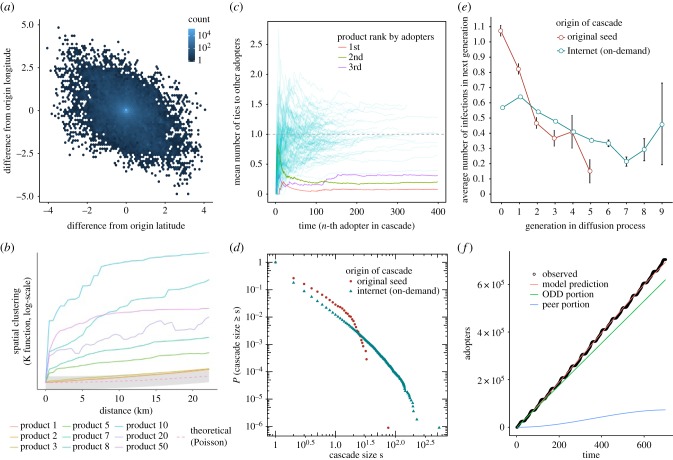
Diffusion dynamics and patterns in the geographical distribution of adopters. (*a*) Relative geographical direction and distance between subsequent adopters. The origin location was centred at 0,0 and the longitude and latitude distance to the next adopting individual are shown. Many adoptions occurred within the same cell tower or over short distances. However, a substantial amount of adoptions occurred between individuals in different parts of the country. (*b*) Geographical spreading pattern for select sample codes of various ranks and a theoretical distribution of random spreading. Plot shows estimates of Ripley's reduced second moment function K(r) from a point pattern in a window of arbitrary shape. Deviations between the empirical and theoretical K curves suggest spatial clustering. The geographical spreading pattern for most codes deviated significantly from the theoretical curve, while codes that spread predominantly via on-demand diffusion (codes ranked 1, 2 and 3) quite closely followed the theoretical curve—indicating geographical independence in adopter locations, which is consistent with on-demand diffusion. (*c*) Degree of ODD and peer-to-peer spreading over time for the 100 codes with the most adopters. The three most popular codes are highlighted (red: most popular; green: second most popular; purple: third most popular). (*d*) Complementary cumulative distribution of cascade sizes for cascades with different origins. (*e*) Difference in infection rate by generation between cascades started from original seeds or Internet adopters. Cascades originating from Internet adopters were deeper and larger in overall size (see electronic supplementary material, table S1). (*f*) Estimated proportion of ODD (green) and social adopters (blue) along with total number of adopters (red) for the most popular code.

How does the nature of ODD relate to the geographical distribution of adopters? To evaluate diffusion dynamics related to geographical spreading, we analysed adopters' geolocation (see electronic supplementary material). We find that spreading along social ties is locally clustered. 240 276 codes spread among individuals within the same cell tower. The median spreading distance was 704 m and the mean distance was 21 km (figure 2*a*). We find significant decay in adoptions with increasing distance (correlation coefficient: −0.19; *p* < 0.001). We next analysed geographical interdependence between adopters (figure 2*b*). We find that most codes spread in a highly geographically clustered fashion. This is especially true for codes that spread to relatively few adopters overall. However, the spreading of the most successful (in terms of total adopters) codes exhibit a pattern that is geographically independent (the spreading of the two most popular codes is not spatially clustered and not significantly different from a random Poisson spreading process). This provides additional support for our claim that non-social spreading is indeed non-social and our claim for two distinct spreading processes: diffusion along social ties (which is geographically clustered), and on-demand diffusion (which is geographically independent). If what we take to be ODD were instead peer-to-peer diffusion but following some unobserved social ties, then we would expect the geographical spreading to be geographically clustered. However, we find no such geographical clustering, providing further evidence for the ODD spreading process.

Having firmly established the presence of ODD, we now ask: how do social and ODD processes differ? We quantified the heterogeneity of the ODD and peer-to-peer spreading process by plotting the average number of ties each adopter had with individuals who adopted earlier (figure 2*c*). Values of one indicate a tree-like spreading pattern where each new adopter is linked to exactly one previous adopter. Values greater than one indicate dense clustering in the social network, such that a new adopter has more than one connection with previous adopters. Values below one indicate a mix of ODD and peer-to-peer spreading where some individuals have no social ties to prior adopters. We find significant heterogeneity, centred on an average of around one. The three most popular codes (highlighted) initially increased in the number of ties—indicating social spreading—but then dropped sharply to close-to-zero ties with prior adopters—indicating ODD spreading. The most popular code spread almost exclusively via ODD adopters who had almost no ties with prior adopters (on average 0.079 ties between each subsequent adopter between the 100th and 1000th adopters). The plot shows not only heterogeneity of ODD and peer-to-peer spreading across codes, but also across time. Early social diffusion turned into non-social diffusion once the product information became available online for on-demand adoption.

To gain more insights into the nature of the diffusion processes and the dichotomy between ODD and peer-to-peer spreading, we analysed the graph structure of invasion trees. The forest of invasion trees contains 100 966 cascades of sizes between two and 535 individuals (figure 2*d*) and depths of up to 10 generations (figure 2*e*). Despite the substantial spread of the most popular code, the largest connected component contains only 1266 adopters. Most codes spread only to a small number of individuals directly connected to the original seed, resulting in many, small connected components. However, the diffusion of the most popular code consisted of many disconnected components, which indicates that the cascade did not exclusively follow existing social ties. Rather, a heterogeneous mix combining ODD and peer-to-peer diffusion characterized the diffusion process. We find many graph components of 2–10 individuals (electronic supplementary material, figure S3). Effectively, ODD spreading creates many disconnected new seeds from which the code then spreads via social ties to several other individuals—thus creating many small clusters of connected adopters. Ultimately, only 30% of adopters remain isolated in the final diffusion graph, indicating that even individuals who initially adopted via the ODD mechanism would subsequently share the code socially. Notable is the absence of individuals that spread a code to many individuals (median = 1; mean = 1.15; max = 53). That is, as predicted by theory [16,34], we do not find super-spreaders.

Next, we explored heterogeneity in diffusion patterns given the origin of the process: cascades started by an individual initially targeted by the experiment, compared to cascades started by on-demand adopters. We find significant differences in cascade size (figure 2*d*) and cascade depth (figure 2*e*) between the two cascade starting points. Cascades started by original seed adopters are significantly smaller (0.80 as large [95% CI: 0.68–0.93]), and significantly shallower (0.90 as deep [95% CI: 0.85–0.95]; electronic supplementary material, table S1) than cascades started by on-demand adopters. While individuals directly targeted with codes managed to diffuse the code to about 1.1 contacts on average, the infection rate decreased with each next generation reaching a maximum cascade depth of six. Individuals starting cascades by on-demand adoption only diffused the code to about 0.6 contacts, thus initially having a lower infection rate than the original seeds. However, starting in the second generation, cascades started by on-demand adopters had a higher infection rate than equal generations in cascades started by original seeds and reached much greater depths (e.g. 0.8 versus 0.6 in the second generation). This suggests that individuals who actively sought product information were in network regions with other susceptible individuals (or somehow had the ability to identify individuals in a heterogeneous population that were better at spreading the code than the individuals reached through original seeds). This allowed ODD to sustain larger and deeper cascades.

To gain deeper understanding into the phenomena of ODD and to check the consistency of our argument that codes posted on the Internet spread in a linear fashion with different levels of fitness, we analysed our data using a simple compartment model. We adapted the model proposed by Hill *et al*. [6]. This approach allows us to better describe the ODD spreading process and to test if the proposed diffusion mechanism is in line with social dynamical processes observed in the population during the experiment. A key feature of the model is its ability to characterize the relative importance of social transmission by quantitatively comparing rates of ODD versus social peer-to-peer adoption. We can thus use the model to estimate the proportion of adopters that are due to on-demand adoption for each code without having to rely on observing network ties. The model extends the classic SIR model to include the possibility for ‘automatic’ non-social infection governed by an additional model parameter *a.* This parameter *a* models the constant rate with which susceptible individuals can autonomously decide to adopt a code by means of active information-seeking (see electronic supplementary material for model details). The motivation for the assumption of a constant background rate of interest in this case is based on the fact that data packages are constantly expiring in this predominantly pre-paid market. Furthermore, the ability to separate social from non-social adoption on the population level allows us to quantify the ‘fitness’ of each voucher code relative to the other voucher codes. We fit the model separately for all codes that accumulated 100 or more adopters using maximum-likelihood and estimate the relative proportion of adopters resulting from social (peer to peer) versus non-social (ODD) diffusion processes (figure 2*f* shows the proportion of ODD and peer-to-peer adopters for the most popular code). Model predictions quantitatively reproduce the actual diffusion data for all observed codes in the experiment and capture the large variation in the relative importance of ODD versus peer-to-peer (electronic supplementary material, table S4). This indicates that the combination of a social diffusion process with spontaneous non-social adoption is a plausible mechanism for the observed spreading dynamic.

We find that the adoption of the most popular code is the combination of 89% ODD adopters and 11% social peer-to-peer adopters. Overall, the four most popular codes are predominantly driven by ODD, while the remaining codes spread predominantly peer-to-peer. We find fitted model parameters for the spontaneous infection parameter *a* indicate systematic inequality in fitness, resulting in vastly different code success. We can use the model to quantify the fitness—i.e. online popularity—of different codes and can quantify how many ODD adopters we expect per day. The code with the highest fitness attracts 2441 ODD adopters per day while the fourth most popular code attracts only four ODD adopters (codes ranked fifth and below attracted less than one ODD adopter per day). These differences in fitness are likely the result of voucher codes posted on different websites with different levels of popularity or position in search engine ranking, ultimately leading to vastly different levels of code success. These results advance our ability to quantify the influence of non-social diffusion mechanisms and the enormous role that ease of discoverability plays in shaping product success.

## 4. Discussion

We used data from two country-scale network experiments to quantify and characterize the adoption of a product (identified through many voucher codes) from active information-seeking behaviour. We find that the product adoption process is characterized by: (a) an interplay of ODD and peer-to-peer diffusion processes interacting simultaneously; (b) linear adoption rates being sustained by a background rate of interest and on-demand information-seeking behaviour; (c) the failure of most voucher codes to spread; (d) structural change in diffusion dynamics when diffusion transitions from peer-to-peer to on-demand; (e) a highly skewed outcome, such that the most popular voucher code receives more adopters than it would in a pure peer-to-peer process; (f) geographical clustering of social diffusion and the independent geographical distribution of on-demand adoption; and (g) longer and deeper cascades of diffusion processes originating from active information-seeking compared to cascades originating from initial seeds.

Understanding the exact pattern, interplay, and prevalence of one social influence process over another is critical to designing successful intervention policies. Diffusion processes that start as viral can change and become driven by on-demand and the ranking of results in online search. The fact that our experiment randomly seeded 70 000 unique voucher codes referencing the same product in the population allowed us to study alternative outcomes in different markets that all start from identical initial conditions. The relative importance of ODD versus viral spreading depends on (a) the background level of interest and the relative fitness of one voucher code over another, and (b) the reproduction ratio of the viral process. In the case of our experiments, the background level of search for information was high relative to the reproduction ratio for the most popular code, but the pattern was reversed for the less popular codes. While small cascades were almost entirely driven by peer-to-peer diffusion, the large cascades were driven almost entirely by ODD.

Furthermore, we find synergies between the two processes in that ODD adopters are more likely to spread the codes in their social network, i.e. ODD allows a vulnerable population to be found. Product diffusion via on-demand information-seeking behaviour can create winner-take-all dynamics driven by the power of rankings on search engines such as Google that would not occur in pure peer-to-peer diffusion. This shows the tremendous impact that rankings in online search have on social influence [20]. Given the ease of posting content to the Internet, the capability for Google and other companies to rapidly index material, and the increased sophistication of search algorithms, we believe that ODD will become an increasingly dominant modality of social influence.

ODD is different from diffusion models that assume ‘automatic’ infection (such as developing obesity [6] or product invention [43]), in that it is influence among strangers, mediated by two steps: (1) posting by the source, and (2) searching by the seeker. As people navigate the Internet more by searching than by browsing, concerns about the influence and potential bias of search engines become crucial [21].

Key design features of our experiment allowed us to isolate effects based on the spread of simultaneously unfolding cascades referencing the same product (that all had randomly seeded starting locations), using an individual-level process rather than aggregate system-level outcomes [20]. Our ability to observe every voucher code's diffusion process provided important insights into population-level diffusion processes. Contrary to traditional broadcast, ODD does not depend on the reach and skill of those running the broadcast campaign, but rather on susceptible individuals who choose to adopt products directly. This choice is driven by their background rate of interests and the ease (and ranking) by which information about products can be found online. The resulting ‘googlization’ leads to significant changes in diffusion processes [20,44].

While ODD dominated the spreading process for the most popular codes in our experiments, our model suggests more generally that product adoption will be guided more by a combination of multiple diffusion processes that happen simultaneously, than by pure regimes. Cascades started by on-demand adopters were larger and deeper than cascades started by originally targeted individuals. This demonstrates substantial heterogeneities in the population regarding their ability to spread products—and demonstrates that ODD can in fact find the better diffusers in the network [45].

Overall, we find consistent results across the two experiments, with linear adoption rates for the most successful codes in both. Furthermore, we find a similar heavy-tailed code distribution popularity, with extreme outlier codes that attracted significantly more adopters than the average code. We contribute to a better understanding of the mechanics of social influence on a global scale, especially in cases where ODD and peer-to-peer influence interact. These results highlight the complex spreading logics in the Internet age [15]. The dominant paradigms of diffusion—peer-to-peer, central source and their combination—are vastly inadequate in understanding the rich diffusion phenomena of the twenty-first century. This paper adds an important logic to our understanding of diffusion—the posting/searching process that allows information and behaviours to rapidly spread among strangers.

These findings, in turn generate numerous additional questions regarding ODD. These future questions include: what are the motivations of people to post information online? When, how, and for which information do people use (the many available) search technologies? How is the ever-increasing sophistication of search engines expanding the scope of the domains subject to ODD?

## Supplementary Material

Supplementary Material

## Endnote

1 Other aspects like the inclusion of product information in a highly visible ‘front page’ or ‘Top 10’ list, and the algorithms of search engines rankings may also affect the shape of product adoption [19,20]. In this paper, we focus on the mixing of peer-to-peer sharing and ODD as these are focal mechanism in this setting.

## Data accessibility

All data required to reproduce key figures and tables in the manuscript is available at http://dx.doi.org/10.7910/DVN/GX2QEZ. The repository also contains our analytic scripts and simulated data. However, raw data (especially for the social network) cannot be released in order to preserve the privacy of individuals.
