# Supplementary material for: Product diffusion through on-demand information-seeking behaviour
Source: J R Soc Interface. 2018 Feb 21;15(139):20170751. doi: 10.1098/rsif.2017.0751 (PMC5832727; doi:10.1098/rsif.2017.0751)
Supplement: Supplementary Material [file rsif20170751supp1.pdf]

Supplementary Material

Product Diffusion Through On-Demand Information Seeking Behavior

Authors: Christoph Riedl,[1,2]* Johannes Bjelland,[3] Geoffrey Canright,[3] Asif Iqbal,[3] Kenth Engø-Monsen,[3] Taimur Qureshi,[3] Pål Roe Sundsøy,[3] David Lazer[1,2]

Published in the Journal of the Royal Society Interface

Affiliations:

[1]Northeastern University, Boston, MA.

[2]Harvard University, Cambridge, MA.

[3]Telenor Research, Oslo, Norway.

*Correspondence to: c.riedl@northeastern.edu.

Supplementary Materials:

Materials and Methods

Experimental Design

We executed both our experiments in a large Asian country. The country is predominantly a prepaid and 2G market; 97% of connections are prepaid and 98% are 2G. In the first half of 2013, smartphones accounted for only 6% of total handset shipments. Given the high reliance on non-data connections, the network can be considered intrinsically dyadic. We partnered with the largest local telecom provider in the country to gain access to a local population of cellphone customers of 50 million customers. The viral product in Experiment 1 (Experiment 2) consisted of an offer for 15MB (60BM) of free data with 15 days (10 days) validity of equal market value in Experiment 1 and Experiment 2 worth approximately USD 40ct. Each viral product consisted of a voucher code represented by a random alphanumeric character sequence (e.g., "A152224YK"). This design prevented that product codes could be guessed. Starting with the whole customer base, we first selected a segment of target customers. That is, individuals who would be eligible to redeem the voucher by demonstrating a minimum level of product affinity. That is, customers needed to be current prepaid subscribers in active status (at least one outgoing SMS in the month prior to the experiment), and they needed to be users of an internet enabled handset but not currently already subscribing to a data plan package (this set of customers constitutes the *target segment*). Within this segment, we randomly assigned 70,000 individuals to receive 70,000 unique voucher codes in Experiment 1 and 48,000 individuals to 48,000 codes in Experiment 2. Experiment 2 used fewer seed products but followed a similar protocol and was conducted about one year after Experiment 1.

Seed individuals were targeted via the following text message:

> "60MB FREE 2G internet Valid 10 Days! To get send A152224YK to **** free and forward & suggest as many friends (pay go or non-data user) to avail same by 13 Jan."

We randomly sampled target individuals to seed products. Seeding the products randomly in the target segment followed the following procedure using information from the full network of connections between all customers based on three month call detail records from the months leading up to the experiment. To test if isolated or clustered seeding would affect the diffusion process we selected initial seed nodes not entirely at random but stratified at random to ensure that some seed nodes would have connections with other seed nodes. Specifically, we sampled seed nodes to be either isolated—having no connection to other nodes in the target segment—or clustered –having connections to other nodes in the target segment and possibly connection to nodes that also received a code. We employed the following sampling procedure. First, we classified all nodes in the target segment as either isolated, a pair, or triangle. Isolated nodes were nodes that had no connection to other individuals in the target segment. Pair nodes were nodes that had a connection to one other node in the target segment. Triangles were three connected nodes within the target segment. Second, among all nodes thus classified, we drew a random sample of 10,000 nodes from the isolated nodes, 20,000 nodes from the pairs, 30,000 nodes from triangle group, and 10,000 nodes entirely at random (from within the target segment). In Experiment 2 we sampled 16,000 from the isolated nodes, and 32,000 nodes from the pairs. These nodes would then each receive a unique product code. In summary, seed nodes have been sampled at random from within the categories. This stratified random assignment procedure ensured that seed nodes had no, one, or two connections with other seed nodes in the target segment who were also seeded a code. Table S1 shows descriptive statistics of key experiment variables for Experiment 1 and 2. Figure S1 shows the number of adopters per product in Experiment 2. Figure S2 shows the cumulative number of adopters over time in Experiment 2.

	Experiment 1	Experiment 2
Products Seeded	70,000	48,000
Individuals Seeded	70,000	48,000
Total Adopters	886,025	328,372
Adopters of most successful product	704,466 (80%%)	116,068 (35%)

Table S1 Descriptive statistics of experimental design for Experiment 1 and 2.

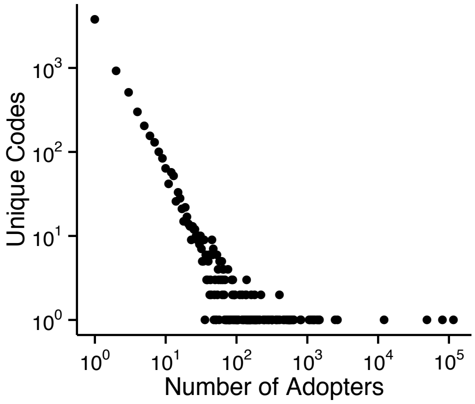

Figure S1 Experiment 2: Number of adopters for each unique product on log-log scale.

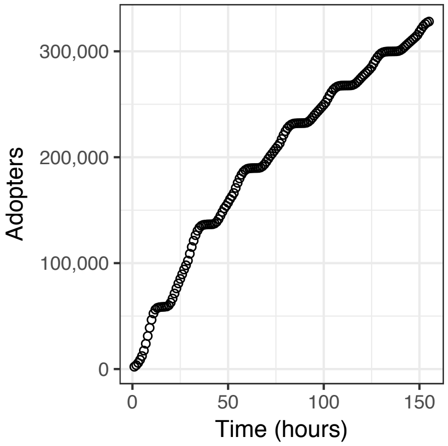

Figure S2 Experiment 2: Cumulative number of adopters aggregated at the full hour.

Full Network

Using call detail records (CDR) from a three-month period following Experiment 2 we constructed the entire social network of all active SIM cards from the carrier we collaborated with. Since voucher codes could only be redeemed by customers of our partner operator, connections outside our operator network are not primarily important. We constructed this as an undirected network without duplicate edges and no self-loops, adding ties between individuals if they either made at least one phone call or sent at least one SMS text message between them. The network consists of 46M nodes and 567M edges. Figure S3 shows the degree distribution of the network.

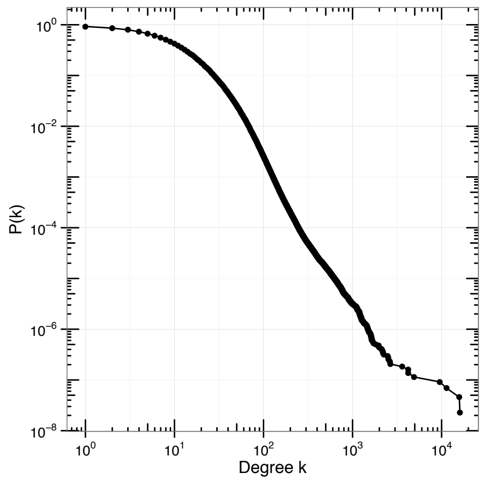

Figure S3 Complementary cumulative degree distribution of nodes in the full social network with N=43m nodes.

Direct Evidence of On-Demand Diffusion

In addition to the empirical evidence discussed in the main body of the manuscript for the presence of ODD, we also sought direct evidence. Following the execution of each experiment, we conducted a systematic search on Google, Facebook, and Twitter for the voucher codes with the highest number of adopters as well as several randomly chosen codes to confirm whether these codes were posted on the internet. We found several codes posted online including the top-three most popular products. The codes appeared mostly on blogs and forum pages under topics such as "cheap deals" and "free data." Figure S4 shows sample screenshot from an online forum in which one of the voucher codes was posted along with instructions on how to redeem the voucher. In Experiment 2 we performed a systematic online search for all voucher codes that garnered more than 100 adopters. We found 69 out of 95 codes online.

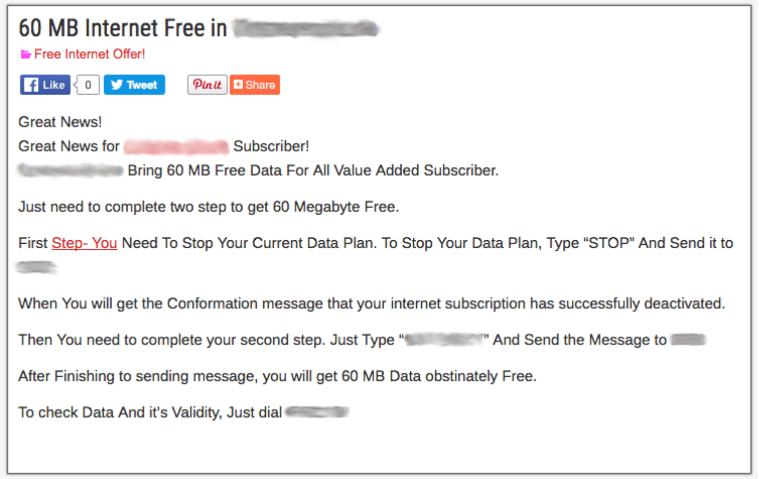

Figure S4 Screenshot of online forum in which voucher code was posted (identity of telecom company obscured).

Stochastic Actor Simulation

We compare the diffusion pattern observed in experiment against simulated canonical stochastic individual contact models. We simulate susceptible-infected (SI), susceptible-infected-recovered (SIR), and Threshold diffusion processes with a wide range of parameter on the full observed social network (46M nodes, 567M edges), randomly seeding 70,000 unique products in the network (the same number of products as in Experiment 1). The SI and SIR model use degree infectivity (*1*). All models rely exclusively on social diffusion processes and we do not allow spontaneous adopters as suggested in the ODD process. We stop the simulation once it reaches the same number of infected individuals as observed in the experiment (or once the epidemic stops spreading). We repeat each simulation ten times to allow variation in initial conditions and analyze the average of the ten simulations. We can characterize the diffusion process using the reproductive number R_0 which is defined as the average number of secondary cases generated by the introduction of a single typical infectious individual (*2*). If the reproduction number $R_0 > 1$, a cascade reaches the tipping point where information reaches a significant fraction of the target population, but if $R_0 < 1$, propagation dies quickly. However, it has been shown that epidemics are possible in scale-free networks with $R_0 < 1$ (*3*). The threshold at which epidemics are possible can be computed as $\frac{\beta}{\mu} \geq \frac{\langle k \rangle}{\langle k^2 \rangle - \langle k \rangle}$ where β is the transmission rate, μ is the recovery rate, and $\langle k \rangle$ and $\langle k^2 \rangle$ are the first and second moments of the degree distribution of nodes in the network. (This computation of the critical value is based on random networks following a configuration model. In real world networks this threshold may be different. To account for this possibility we perform robustness test using slightly different values above and below the critical value computed above.) To calibrate the model, we computed the typical infection rate of early individuals (transmission rate of seed nodes who adopted) as $\beta = 0.029$, the recovery rate μ is not observed. Substituting known values into the above equation, we can compute $\mu = 0.456$. For robustness, we perform simulations using different values of β and μ at or above the epidemic threshold (if values are below the threshold the epidemic is not possible and would die out).

We perform several robustness tests of our simulations. First, the critical value of the diffusion process computed above may be different in in real world networks. Figure S5 shows the distribution of cascade sizes resulting from simulations of the SIR model with parameter combinations for β and μ above and below the critical value computed above. Using infection rates above or below the critical value does not substantively alter our conclusions. Second, we may not observe all social ties, e.g., because individuals communicate using Facebook or other messaging services. Hence, we simulate the diffusion process on modified, denser networks to which we add random edges following the approach suggested by Adiga et al. (*4*). Specifically, we created a new modified network for each simulation run in which we take the observed network as the basis but then add 5% or 10% additional random edges using, either uniformly or degree assortative (Figure S6). Simulating the diffusion process on these denser networks does not substantively alter our conclusions. Figure S7 shows the distribution of cascade sizes for threshold models using different thresholds. The distribution of cascade sizes resulting from simulations of the threshold model also does not fit the observed distribution. A possible limitation of this robustness test is that we have no measure for the number of unobserved ties. As such adding 5% or 10% of ties may be too many or too few.

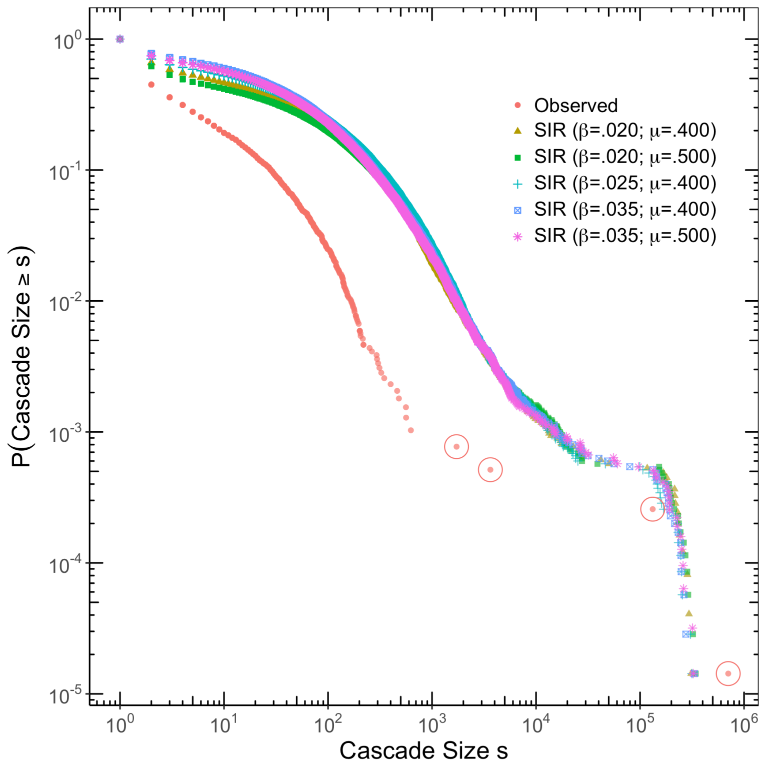

Figure S5 Robustness tests simulating the SIR model with parameter values for $\boldsymbol{\beta}$ and $\boldsymbol{\mu}$ above and below the critical value.

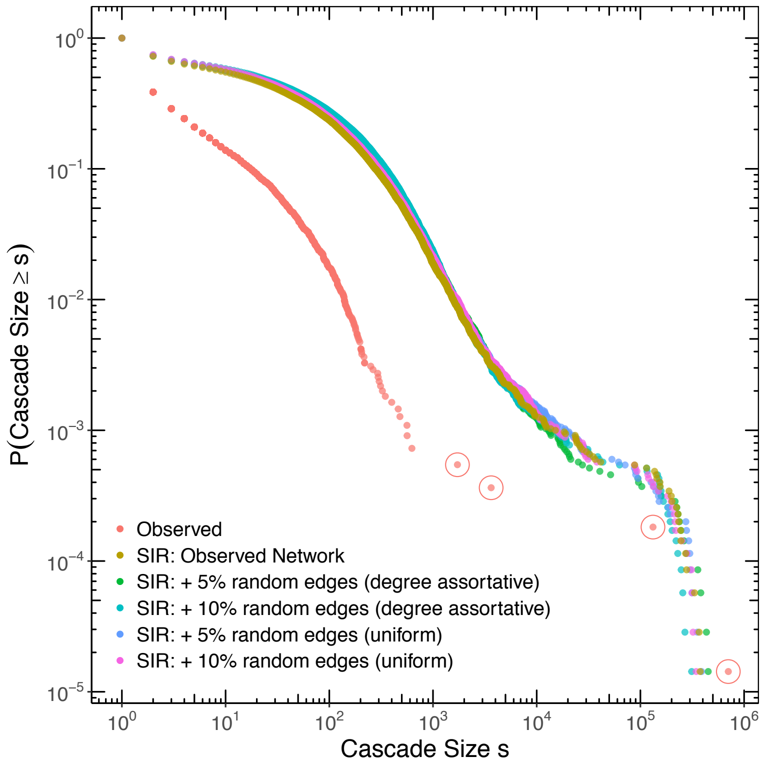

Figure S6 Simulation of SIR model on noisy networks which contain additional random ties.

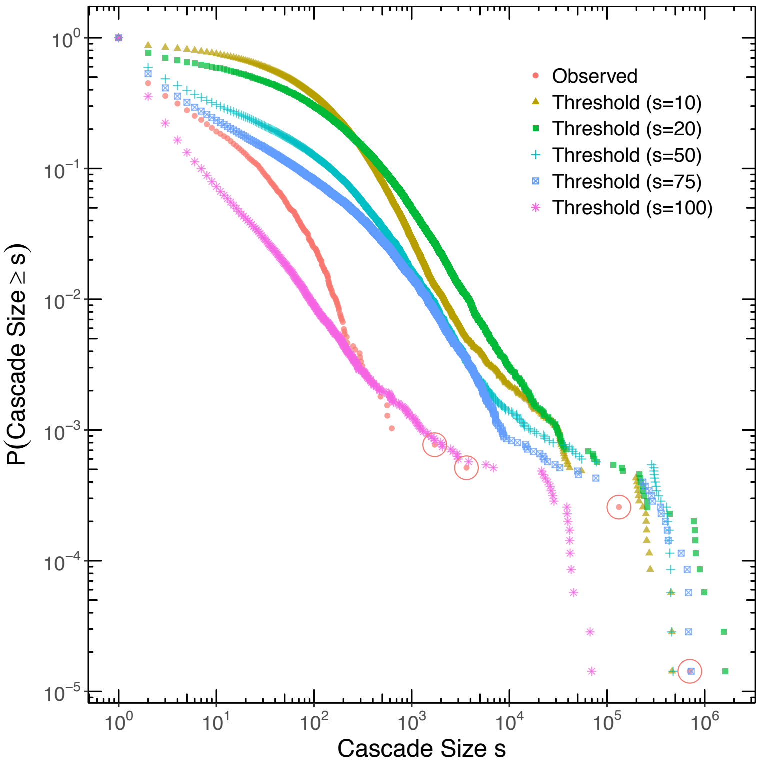

Figure S7 Distribution of cascade sizes for threshold models using different thresholds.

We then computed the percent of adopters who adopted the most popular product in each of the 10 simulation runs we did for the different models and parameter configurations (Table S2 show the average and 95% confidence interval for select models). In the experiment, 80% of adopters adopted the most popular product. In each of the simulations, across the different models we find significantly fewer adopters adopt the most popular product. Typically, only around 30% of the adopters adopt the most popular product in the SIR and SI models and around 54% in the threshold models—significantly fewer than the 80% observed in the experiment. We also perform a statistical test whether the distribution of cascade sizes from any of the simulations is drawn from the same distribution as the observed distribution using a two-sample Kolmogorov-Smirnov test. The test rejects the null hypothesis that the data are drawn from the same distribution (comparing the observed distribution to the result of the threshold model (s=75): D = 0.08, p-value < 2.2e-16).

	Percent Adopters of Most Popular Product	95% CI
Observed	80%	
SIR ($\beta = .029, \mu = .456$)	32%	[27% – 36%]
SIR (dito, with 10% random edges (da))	26%	[23% – 29%]
SI ($\beta = .029$)	26%	[24% – 29%]
Threshold ($s = 75$)	57%	[46% – 69%]
Threshold ($s = 100$)	51%	[44% – 58%]

Table S2 Comparison of adopters of the most popular product.

Geolocation

Using call detail records we can determine which cell tower a customer is "logged in" most of the time. We designate this the "home cell" of an individual. Using infrastructure data about geographic distribution of cell towers we can thus determine the "home geolocation" of each customer and thus investigate patterns in geographic spreading. Since the resolution of our geographic data is at the level of the cell tower, spreading distance between individuals who are associated with the same home cell are recorded with a distance of 0km.

Invasion Trees Analysis

One challenge in the analysis of invasion trees is that while we observe (1) the social network of ties between individuals, (2) which individual adopted a product, and (3) the exact time of product adoption, we do not directly observe along which ties the products spread. We imputed invasion trees from observed network and diffusion data as follows. If an individual *A* has a network tie with another individual *B* where *B* adopted the same code prior to *A* adopting the same code, we add a tie between *A* and *B* in the invasion tree. If *A* has ties with multiple individuals *B* that adopted the same code previously, we add a tie to the *B* individual with the strongest network tie (based on voice minutes). In case of multiple *B* with identical edge weights, we selected a random *B*. Our analysis makes the simplifying assumption that individuals can adopt a product only by the influence of a single neighbor or via information seeking. However, there may be multiple stimuli of social influence from several neighbors. That is, an individual may be exposed to several different product codes from different neighbors in the social network out of which the individual would then choose one to adopt. In this sense, the emerging cluster of linked individuals adopting a product is not necessarily a tree but may contain loops.

To test for structural differences of invasion trees resulting from cascades with different origins (ODD vs. peer-to-peer), we perform Poisson regression analysis on key statistics of the trees: their size (number of nodes), width, and depth (Table S3). Given that cascades start at different points in time, it is imperative to control for start time (cascades that start later, have less time to propagate till the end of the experiment and are thus expected to be systematically smaller). We compute clustered standard errors that are clustered by voucher code. The coefficient of *Start Time* is statistically significant ($p < .001$) and negative in all three models indicating, as expected, that cascades that started later were generally small, narrower, and shallower. We find that cascades started from the original seed nodes are significantly smaller ($p < .01$) and also significantly shallower ($p < .001$) but not necessarily wider (not significant). This supports the conclusion that cascades started by on-demand adopters were larger, and deeper than those started by original seeds.

Dependent Variable:	Tree Size	Tree Width	Tree Depth
Intercept	1.021***	0.466***	0.496***
	(0.082)	(0.048)	(0.031)
Source: Original	−0.233**	−0.082	−0.105***
	(0.081)	(0.047)	(0.030)
Start Time (h)	−0.004***	−0.002***	−0.002***
	(0.000)	(0.000)	(0.000)
AIC	1,884,709.380	1357,309.742	1,329,268.587
Log Likelihood	−942,351.690	−678,651.871	−664,631.293
Deviance	655,702.037	190649.017	127,053.203
Num. obs.	562,000	562,000	562,000

$^{***}p < 0.001$, $^{**}p < 0.01$, $^{*}p < 0.05$

Table S3 Comparison of structural differences of invasion trees (Poisson regression, clustered standard errors reported in parentheses (clustered by voucher code)).

Multiple-Infections and Collisions

By design of the viral product, every individual was allowed to adopt only one product (i.e., redeem only a single voucher code). Conceptually, this is equivalent to the notion of acquired immunity and cross-immunity in the context of multi-pathogen competition (*5*). Since we observe the full social graph of social connections among all adopters, we can quantify collision rates, i.e., instances in which individuals are exposed to two or more different products from which they can chose to adopt at most one (Fig. 1F). Insights into competition in multi-product adoption requires precise information about the social graph and would not be possible with aggregate time series data. We find that the interface of the propagation of the multiple epidemics, i.e., the set of individuals that are reached by two or more products, is substantial: 16% of adopting individuals have two or more unique products in their social network. However, the likelihood of collisions is not homogeneous such that some voucher codes experience many more collisions with competing voucher codes than others. We find collisions range from no collisions to 100% (mean=22%; SD=28%). The collision rate naturally increases over the course of the experiment. As more and more individuals adopt, the prevalence of products in the population increases and collision rate increases (Figure S8).

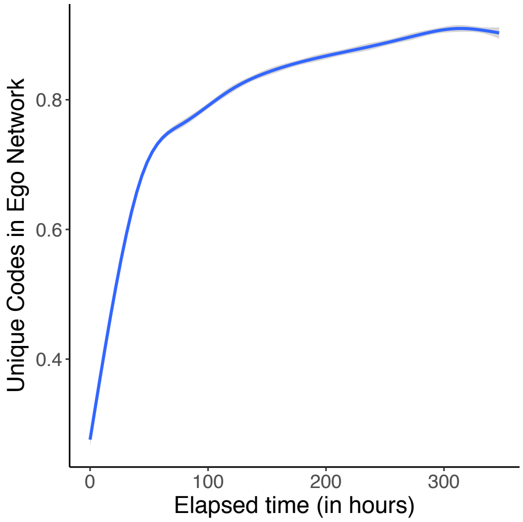

Figure S8 The collision rate rapidly increases in the first hours of the spreading process.

Time Series Analysis

To test for structural change in the temporal pattern in number of adopters for the most popular product, we performed additional time series analysis. We consider the time series of product adopters of the most popular product. This analysis in particular would reveal whether there is a structural change in the number of adopters over time. We would expect such a structural change if indeed the spreading changed from an exponential growth regime (as would be expected during a period of social peer to peer spreading) to a linear growth regime (as would be expected during non-social on-demand spreading). We first decompose the raw data of the time series into a cyclic (diurnal) pattern, the overall trend of increase in adopters, and an error measure. The decomposed trend shows a steep increase at the beginning of the first day, followed by a relatively steady pattern thereafter (Figure S9). To test whether the structural change is significant or within the expected random fluctuation, we perform a F-stat based test for structural stability following the procedure suggested by Andrews (*6*). We use the implementation of the test provided by the strucchange package in R (*7*). This test computes a F statistic (Chow test statistic) for every potential change point by fitting an OLS model for the observations before and after the potential change point and the error sum of squares (ESS). The test confirms a structural change during day one ($p < .001$; Figure S10).

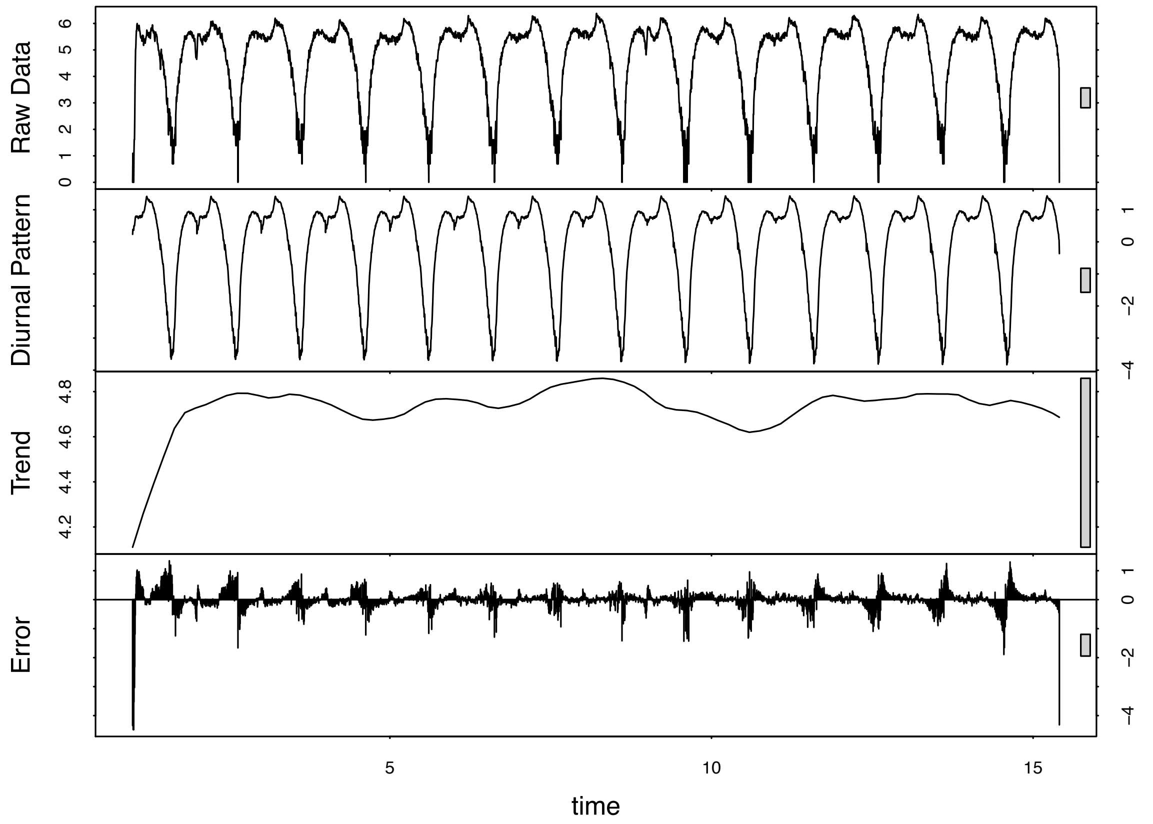

Figure S9 Time series analysis of number of the logged number of new adopters of the most popular product per six minutes time window. Top panel: raw data. Top-middle panel: diurnal pattern. Bottom-middle: Trend after extracting diurnal pattern. Bottom: remainder (error).

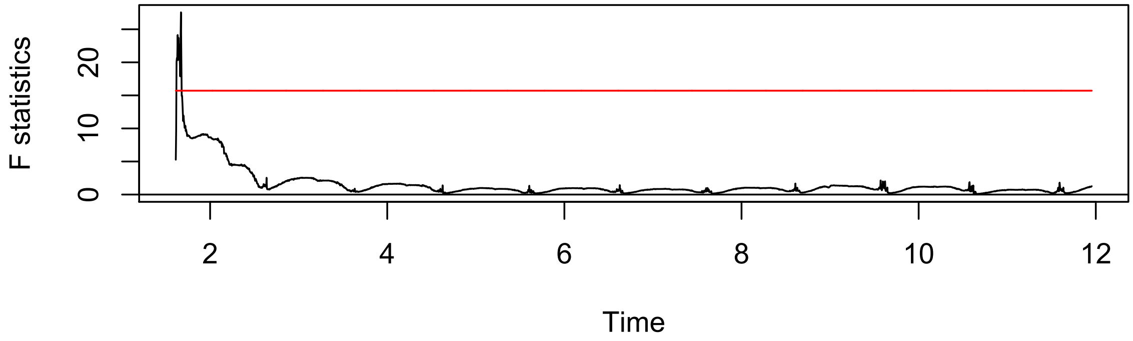

Figure S10 Structural change test confirms change from exponential to linear growth during day one (points above the red line indicate statistical significance).

Deterministic Compartment Model

We adapt the deterministic compartment model proposed by Hill et al. (*8*) to describe the ODD spreading process. Albeit simple, a deterministic compartment model is useful to help understand mechanisms and patterns of diffusion processes (*9*). In the model individuals are classified as occupying one of three states: “susceptible” (S) individuals do not have the disease, “infected” (I) individuals who have the disease and can infect others, and “recovered” (R) individuals who have recovered and become immune to further infection. Contrary to other behavior, trends, and health states which can occur repeatedly in an individual (*8*), in our case individuals can adopt only one product and are then immune to further infection. While an individuals can continue to spread the product any time until the end of the campaign and never become “recovered” in a technical sense we find that, empirically, individuals either spread the product very quickly or not at all. We find that 65% of socially spread product adoptions happen within 24 hours. Consequently, the susceptible-infected-recovered (SIR) model is more appropriate than the susceptible-infected-susceptible (SIS) model used by Hill et al. which allows repeated infection. The rate of this disease transmission from susceptible to infected is defined as ß. Individuals recover at a constant rate g, which is independent of their contacts with susceptible or infected. This can be interpreted as infected individuals ceasing to be infectious. This is particularly relevant in our empirical setting as individuals are likely to forget about or lose interest in the product. Furthermore, individuals in the population can autonomously become infected at a constant rate a, independent of contacts with infected individuals (see, e.g., (*10*) for another approach to add exogenous/spontaneous infection rate to information diffusion models called the Bass model). This constant rate a is likely to differ between products such that product codes that are posted on more popular website or are ranked higher in the search results would garner higher rates of autonomous adopters. The differential equations for the deterministic compartment model are given in equation S1. We then fit the model separately using maximum likelihood to all products that accumulated 100 or more adopters and estimate the relative proportion of adopters resulting from social (peer to peer) versus non-social (ODD) diffusion processes (Table S4).

$$
\begin{aligned}
dS/dt &= -\beta SI - aN \\
dI/dt &= \beta SI + aN - gI \\
dR/dt &= gI \\
S + I + R &= N
\end{aligned}
\tag{S1}
$$

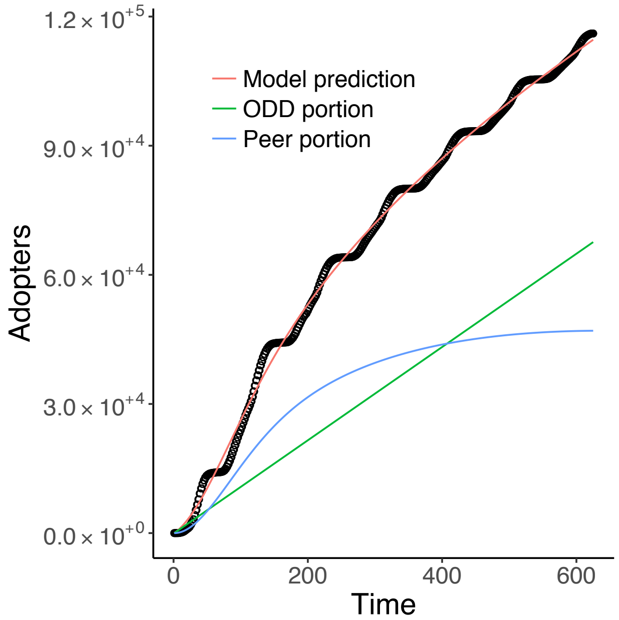

Figure S11 Estimated proportion of ODD (green) and social adopters (blue) along with total number of adopters (red) for most popular product in Experiment 2.

Rank	Total	β	g	a	RMSE	ODD	Peer	ODD per Day
All	886,025	1.07E-12	4.41E-06	2.70E-05	7,391.44	98%	2%	2,438
1	704,466	1.29E-09	2.18E-06	1.94E-05	5,569.13	89%	11%	1,750
2	132,295	5.58E-10	1.57E-05	4.18E-06	1,292.08	99%	1%	376
3	3,638	8.06E-07	5.04E-07	7.08E-08	57.66	65%	35%	4
4	1,720	4.19E-05	1.43E-01	3.94E-08	20.12	74%	26%	4
5	625	9.88E-06	2.29E-10	4.84E-09	18.15	27%	73%	< 1
10	399	2.59E-05	2.11E-03	1.86E-09	10.82	16%	84%	< 1
20	218	4.34E-05	2.66E-03	2.04E-09	4.40	31%	69%	< 1
75	119	7.67E-05	8.20E-04	1.04E-09	2.61	27%	73%	< 1

Table S4 Fitted model parameters values for fitted parameter β, g, and a for select products with fitting error (RMSE). Products with more than 1,000 adopters are predominantly driven by ODD adoption. Product Rank 1 has the highest fitness a leading to an estimated 1,772 ODD adopters per day.

References

1. P. C. Staples, E. L. Ogburn, J.-P. Onnela, Incorporating Contact Network Structure in Cluster Randomized Trials. *Sci. Rep.* **5**, 1–12 (2015).
2. M. J. Keeling, P. Rohani, *Modeling Infectious Diseases in Humans and Animals* (Princeton University Press, Princeton, NJ, 2008).
3. R. Pastor-Satorras, A. Vespignani, Epidemic spreading in scale-free networks. *Phys. Rev. Lett.* **86**, 3200–3203 (2001).
4. A. Adiga, C. J. Kuhlman, H. S. Mortveit, A. K. S. Vullikanti, Sensitivity of Diffusion Dynamics to Network Uncertainty. *J. Artif. Intell. Res.* **51**, 207–226 (2014).
5. C. Poletto *et al.*, Characterising two-pathogen competition in spatially structured environments. *Sci. Rep.* **5**, 1–9 (2015).
6. D. W. K. Andrews, Tests for parameter instability and structural change with unknown change point. *Econometrica*. **61**, 821–856 (1993).
7. A. Zeileis, F. Leisch, K. Hornik, C. Kleiber, strucchange: An R Package for Testing for Structural Change in Linear Regression Models. *J. Stat. Softw.* **7**, 1–38 (2002).
8. A. L. Hill, D. G. Rand, M. A. Nowak, N. A. Christakis, Infectious disease modeling of social contagion in networks. *PLoS Comput. Biol.* **6** (2010), doi:10.1371/journal.pcbi.1000968.
9. R. M. Anderson, R. M. May, B. Anderson, *Infectious diseases of humans: dynamics and control* (Oxford University Press, 1992).
10. F. M. Bass, A New Product Growth for Model Consumer Durables. *Manage. Sci.* **15**, 215–227 (1969).
